# Early Pixel Value Ratios to Assess Bone Healing During Distraction Osteogenesis

**DOI:** 10.3389/fbioe.2022.929699

**Published:** 2022-07-12

**Authors:** Qi Liu, Haibo Mei, Guanghui Zhu, Ze Liu, Hongbin Guo, Min Wang, Jieyu Liang, Yi Zhang

**Affiliations:** ^1^ Department of Orthopaedics, Xiangya Hospital, Central South University, Changsha, China; ^2^ National Clinical Research Center for Geriatric Disorders, Xiangya Hospital, Central South University, Changsha, China; ^3^ Department of Pediatric Orthopedics, Hunan Children’s Hospital, Pediatric Academy of University of South China, Changsha, China; ^4^ Department of Endocrinology, Xiangya Hospital, Central South University, Changsha, China

**Keywords:** distraction osteogenesis, bone lengthening, pixel value ratio, X-ray, external fixator

## Abstract

**Background:** Distraction osteogenesis (DO) is an approach for bone lengthening and reconstruction. The pixel value ratio (PVR), an indicator calculated from X-ray images, is reported to assess the final timing for the external fixator removal. However, the early PVR and its potential influencing factors and the relationship between the early PVR and clinical outcomes are rarely discussed. Therefore, this study was employed to address these issues.

**Methods:** A total of 125 patients with bone lengthening were investigated retrospectively. The early PVR of regenerated bone was monitored in the first 3 months after osteotomy. The potential effect of sex, chronological age, BMI, lengthening site, and involvement of internal fixation during the consolidation period was analyzed. Moreover, the associations of the healing index (HI) and lengthening index (LI) with early PVR were also investigated.

**Results:** The early PVRs were 0.78 ± 0.10, 0.87 ± 0.06, and 0.93 ± 0.06 in the first 3 months after osteotomy, respectively. Moreover, the PVR in juvenile was significantly higher than that in adults in the first 3 months after osteotomy (0.80 ± 0.09 vs. 0.74 ± 0.10; *p* = 0.008), (0.89 ± 0.06 vs. 0.83 ± 0.06; *p* = 0.018), and (0.94 ± 0.05 vs. 0.87 ± 0.05; *p* = 0.003). In addition, the PVR in males was significantly higher than that in females in the first month after osteotomy (0.80 ± 0.09 vs. 0.76 ± 0.10; *p* = 0.015), and the PVR in femur site was significantly higher than that in the tibia site in the second and third months after osteotomy (0.88 ± 0.07 vs. 0.87 ± 0.06; *p* = 0.015) and (0.93 ± 0.06 vs. 0.92 ± 0.06, *p* = 0.037). However, the BMI and involvement of the internal fixator during the consolidation period seem to not influence the early PVR of regenerated callus during DO. Interestingly, the early PVR seems to be moderately inversely associated with HI (mean = 44.98 ± 49.44, r = -0.211, and *p* = 0.029) and LI (mean = 0.78 ± 0.77, r = -0.210, and *p* = 0.029), respectively.

**Conclusion:** The early PVR is gradually increasing in the first 3 months after osteotomy, which may be significantly influenced by chronological age, sex, and the lengthening site. Moreover, the early PVR of callus may reflect the potential clinical outcome for DO. Our results may be beneficial to the clinical management of the subjects with bone lengthening.

## 1 Introduction

Distraction osteogenesis (DO) is an approach for bone lengthening and reconstruction. Generally speaking, the regeneration system in living tissue is activated under a physiological continuous, stable, and slow distraction force: the bone and its attached muscles, fascia, blood vessels, and nerve tissue grow synchronously. This technique is utilized to treat severely damaged limb tissues and complicated orthopedic disorders (Malkova and Borzunov, 2021). Basically, DO is an effective treatment for significant bone defects, limb shortening, bone non-union, limb deformities, and neurovascular skin injuries (Shchudlo et al., 2017).

Currently, a variety of evaluation methods have been employed to monitor bone lengthening during DO, including standard radiography (X-ray), dual-energy X-ray absorptiometry (DXA), quantitative computed tomography (QCT), ultrasound, biomechanical evaluation, and biochemical markers (Tesiorowski et al., 2005; Babatunde et al., 2010). In general, the cost and radiation of QCT and DXA are high, and the scope of their application is also limited (Babatunde et al., 2010; Engelke et al., 2013; Anna et al., 2021). Moreover, ultrasound cannot penetrate the cortex of mature bone, and the limb line of force is not intuitive enough to be presented either (Eyres et al., 1993). Biomechanical testing is usually considered for laboratory fundamental research (Floerkemeier et al., 2005; Ishimoto et al., 2011). Nevertheless, X-ray is the most common choice for being inexpensive and convenient. However, this evaluation is relatively subjective (relied on the experience of clinicians) (Starr et al., 2004; Anand et al., 2006). Therefore, an objective quantitative assessment based on X-ray is quite needed.

The pixel value (PV) is an assessment of the bone mineral density in pixels. On this basis, the pixel value ratio (PVR) is calculated by comparing the PV of regenerated bone with that of the adjacent bone (Bafor et al., 2020). Hazra et al. (2008) found that there was a good correlation between the BMD (bone mineral density) ratio and PVR, which suggested decent reliability of the PVR. The density of the regenerated bone increases with healing and leads to a higher PVR (approach to 1). Furthermore, the PVR can be calculated in the clinical setting without any additional expense or radiation for the patient, which makes it a potentially attractive method to objectively measure the status of regenerated bone healing. Therefore, PVR is a quantifiable, convenient and reliable evaluative indicator to monitor the formation of newborn callus.

To the best of our knowledge, PVR is mainly utilized to assess the maturity of the late callus and to confirm the timing to remove the external fixator (Hazra et al., 2008; Zhao et al., 2009; Song et al., 2012; Vulcano et al., 2018; Bafor et al., 2020). However, the early PVR of the callus during DO is rarely discussed. Importantly, the early PVR can assess the progress of callus maturation, which may be important for deciding the lengthening speed during the early DO stage (slow down or speed up the lengthening). Moreover, it has been reported that age, sex, BMI, lengthening site, and the involvement of internal fixation during the consolidation period may significantly affect osteogenesis (Mehta et al., 2011; Sun et al., 2011; Ko et al., 2019; Zak et al., 2021), which may further exert impact on PVR as a consequence. On the other hand, the lengthening index (LI) is the number of months required to achieve 1 cm lengthening, whereas the healing index (HI) is calculated as the duration of complete consolidation in days divided by the length gained in centimeters. Both LI and HI are reliable indicators of the bone healing potential (Koczewski and Shadi, 2013; Wright et al., 2020). However, the associations of LI and HI with early PVR have never been explored yet. Therefore, this study was employed to investigate: 1) the early PVR of the callus in bone lengthening; 2) the potential influencing factors for the early PVR; and 3) the associations of LI and HI with the early PVR of the callus.

## 2 Materials and Methods

### 2.1 Study Design and Patients

**FIGURE 1:**
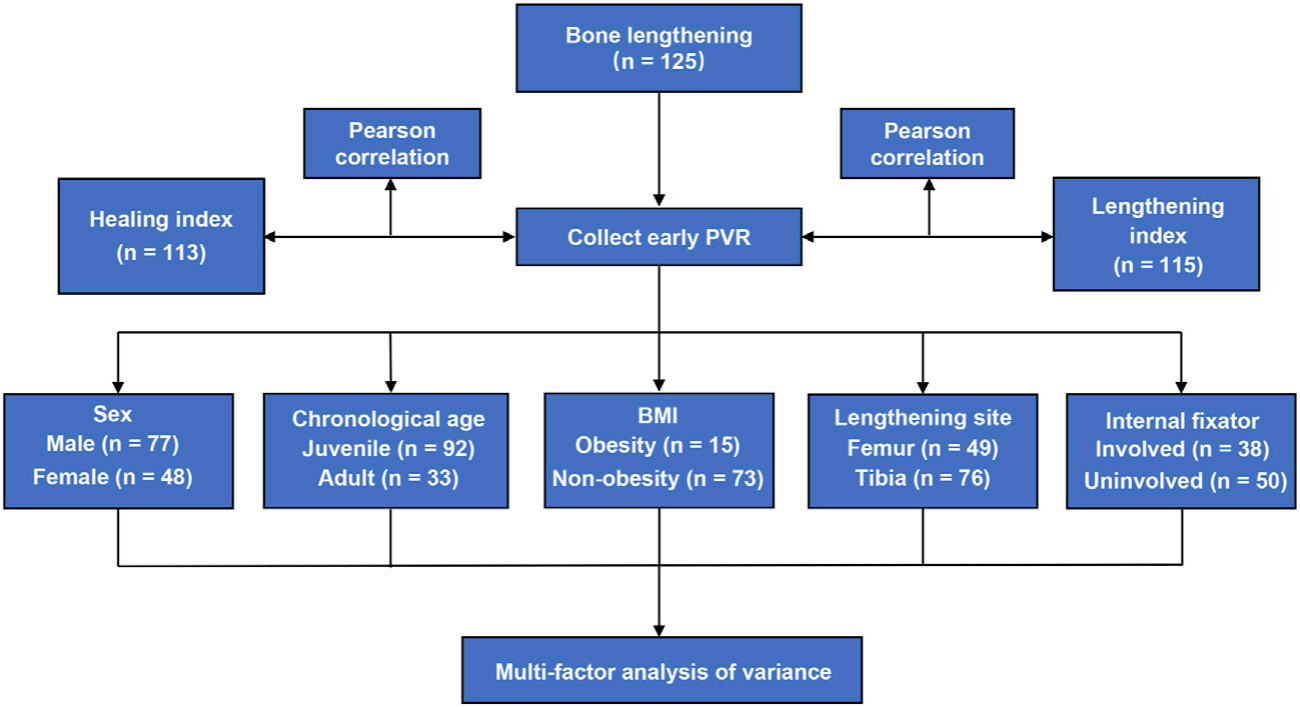
Flowchart for the study design of this study.

This study was approved by the ethics committee of the Xiangya Hospital of Central South University. We retrospectively reviewed the clinical and imaging data of patients who completed bone lengthening in the Xiangya Hospital of Central South University and Hunan Children’s Hospital from January 2010 to December 2021. The inclusion criteria were as follows: 1) lower limb lengthening by using the Ilizarov technique; 2) primary surgery; and 3) patients with successful bone lengthening. The exclusion criteria were as follows: 1) patients with the bone non-union or delayed union; 2) patients with a skeletal disorder affecting the healing index, for example, congenital pseudarthrosis of the tibia; and 3) patients with missing follow-up data. Finally, a total of 125 subjects were recruited for our study. A chart for the study design has been shown in Figure 1.

### 2.2 Surgical Method

All patients were operated on by experienced surgeons, and the Ilizarov technique was used for bone lengthening in the femur and tibia. The patients were subjected with or without the involvement of an internal fixator during the consolidation period (after reaching the final length) randomly. As a consequence, 72 patients were kept with an external fixator until the callus was completely healed, whereas 53 patients were replaced with an internal fixator during the consolidation period. The distraction was initiated 1 week after the osteotomy (at the speed of 0.75–1 mm per day). Then, the patients were examined by X-ray monthly. The conditions to end the bone lengthening and remove the external fixator are listed as follows (patients kept with external fixator): 1) bridging callus is shown on three of the four cortices based on the anteroposterior and lateral X-ray photos of the extension segment; 2) the fixation time is generally in line with the average extension index (the total fixation time of the external fixator is the average time of soft callus consolidation calculated from the date of lengthening. Each 1 cm is fixed for 1 month, called the average extension index); and 3) no abnormal feeling of weight-bearing after loosening the nut (Iobst et al., 2017).

### 2.3 Pixel Value Ratio Measurement Based on X-Ray

**FIGURE 2:**
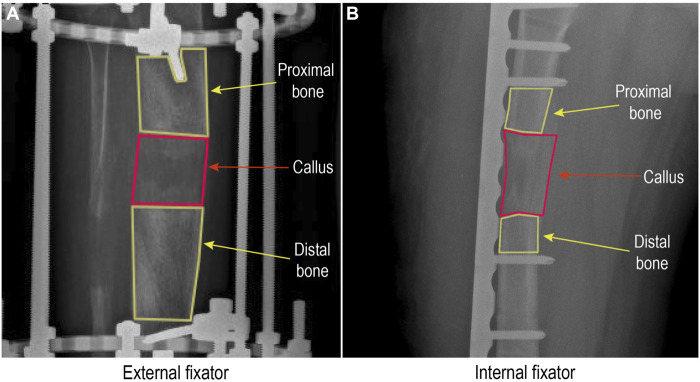
Pixel value assessment from a radiograph in a picture archiving and communication system.

The X-ray image measurement tool of the picture archiving and communication system (PACS) was employed to depict the regenerated bone area and its distal and proximal normal bone areas (Figure 2). In order to improve the accuracy of the PVR, the part of the metal bar was rigorously avoided in the targeted area. Then, the ratio of the regenerated bone PV to the average value of the distal and proximal normal bone PV was calculated. The higher PVR (approach to 1) indicates that the regenerated callus is closer to the adjacent normal bone, whereas the lower PVR reflects a lower immaturity (Zhao et al., 2009). The formula for calculating PVR is as follows:

PVR =Regenerated bone pixel value(Distal normal bone pixel value + Proximal normal bone pixel value )÷2.                 

### 2.4 Potential Influencing Factors for the Early Pixel Value Ratio of the Callus During Distraction Osteogenesis

The overall PVR is analyzed first. Then, the subjects were divided into several subgroups according to sex, chronological age, BMI, lengthening site, and the involvement of the internal fixator during the consolidation period, respectively.

### 2.5 Associations of the Healing Index and Lengthening Index With the Early Pixel Value Ratio of the Callus

The LI is the number of months required to achieve 1 cm lengthening, whereas the HI is calculated as the duration of complete consolidation (three cortices in the distraction callus) in days divided by the length gained in centimeters (Koczewski and Shadi, 2013; Wright et al., 2020). The mean of the HI and LI in these patients and the associations of HI and LI with the early PVR of the callus have been analyzed.

### 2.6 Statistical Analysis

All the analyses were performed by using the SPSS 26.0 version. The mean value and standard deviation (SD) of the PVR for the first 3 months were calculated. The PVR differences according to sex (male vs. female), chronological age (juvenile: under 18 years old vs. adult: 18 years or older), BMI (non-obesity vs. obesity), lengthening site (femur vs. tibia), and involvement of the internal fixator were assessed by a multi-factor analysis of variance. The associations of the HI and LI with the early PVR of callus were assessed by Pearson’s correlation coefficient. *p* < 0.05 was regarded as statistically significant.

## 3 Results

### 3.1 Pixel Value Ratio in Subjects With Bone Lengthening Is Gradually Increasing During the First Three Months After Osteotomy

**FIGURE 3:**
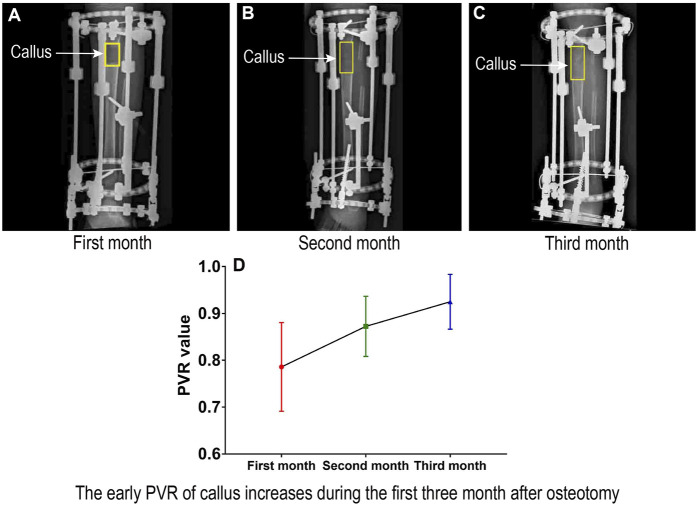
Early PVR of the regenerated callus during distraction osteogenesis.

A total of 125 patients were recruited for this analysis. The PVRs in subjects with bone lengthening were 0.78 ± 0.10, 0.87 ± 0.06, and 0.93 ± 0.06 in the first 3 months, respectively (Table 1). Obviously, the PVR increased gradually during the first 3 months after osteotomy (Figure 3).

**TABLE 1:** Early PVR value of the regenerated callus during distraction osteogenesis.

	First month (*n* = 118)	Second month (*n* = 107)	Third month (*n* = 102)	*p*-value
PVR value (mean ± SD)	0.78 ± 0.10	0.87 ± 0.06	0.93 ± 0.06	<0.001

### 3.2 Subgroup Analysis for the Pixel Value Ratio

#### 3.2.1 Subgroup Analysis for the Pixel Value Ratio Based on Sex

A total of 77 male and 48 female subjects were recruited for this analysis. The PVR in males with bone lengthening was significantly higher than that in females in the first month after osteotomy (0.80 ± 0.09 vs. 0.76 ± 0.10; *p* = 0.015). However, there was no significant difference in the second and third month (0.87 ± 0.07 vs. 0.87 ± 0.06; *p* = 0.690), (0.93 ± 0.06 vs. 0.92 ± 0.06; *p* = 0.504) (Table 2).

**TABLE 2:** Subgroup analysis for the early PVR based on sex.

PVR value (mean ± SD)	Sex		*p*-value
	Male (*n* = 77)	Female (*n* = 48)	
First month	0.80 ± 0.09	0.76 ± 0.10	0.015
Second month	0.87 ± 0.07	0.87 ± 0.06	0.690
Third month	0.93 ± 0.06	0.92 ± 0.06	0.504

#### 3.2.2 Subgroup Analysis for the Pixel Value Ratio Based on Chronological Age

A total of 92 juvenile and 33 adult subjects were recruited in this analysis. The PVR in juveniles with bone lengthening was significantly higher than that in adults in the first 3 months after osteotomy (0.80 ± 0.09 vs. 0.74 ± 0.10; *p* = 0.008), (0.89 ± 0.06 vs. 0.83 ± 0.06; *p* = 0.018), and (0.94 ± 0.05 vs. 0.87 ± 0.05; *p* = 0.003) (Table 3).

**TABLE 3:** Subgroup analysis for the early PVR based on chronological age.

PVR value (mean ± SD)	Chronological age		*p*-value
	Juvenile (*n* = 92)	Adult (*n* = 33)	
First month	0.80 ± 0.09	0.74 ± 0.10	0.008
Second month	0.89 ± 0.06	0.83 ± 0.06	0.018
Third month	0.94 ± 0.05	0.87 ± 0.05	0.003

#### 3.2.3 Subgroup Analysis for the Pixel Value Ratio Based on BMI

A total of 15 obese and 73 non-obese subjects were recruited for this analysis. There was no significant difference in the PVR between the obese and non-obese subjects with bone lengthening in the first 3 months after osteotomy (0.76 ± 0.11 vs. 0.79 ± 0.10; *p* = 0.854), (0.87 ± 0.07 vs. 0.88 ± 0.06; *p* = 0.116), and (0.90 ± 0.06 vs. 0.94 ± 0.05; *p* = 0.154) (Table 4).

**TABLE 4:** Subgroup analysis for the early PVR based on BMI.

PVR value (mean ± SD)	BMI		*p*-value
	Obesity (*n* = 15)	Non-obesity (*n* = 73)	
First month	0.76 ± 0.11	0.79 ± 0.10	0.854
Second month	0.87 ± 0.07	0.88 ± 0.06	0.116
Third month	0.90 ± 0.06	0.94 ± 0.05	0.154

#### 3.2.4 Subgroup Analysis for the Pixel Value Ratio Based on the Lengthening Site

A total of 49 femoral and 76 tibial site subjects were recruited for this analysis. The PVR in the femur site was significantly higher than that in the tibia site in the second and third months after osteotomy (0.88 ± 0.07 vs. 0.87 ± 0.06; *p* = 0.015) and (0.93 ± 0.06 vs. 0.92 ± 0.06; *p* = 0.037). However, there was no difference in the first month (0.80 ± 0.10 vs. 0.76 ± 0.09; *p* = 0.349) (Table 5).

**TABLE 5:** Subgroup analysis for the early PVR based on the lengthening site.

PVR value (Mean ± SD)	Lengthening site		*p* value
	Femur (*n* = 49)	Tibia (*n* = 76)	
First month	0.80 ± 0.10	0.76 ± 0.09	0.349
Second month	0.88 ± 0.07	0.87 ± 0.06	0.015
Third month	0.93 ± 0.06	0.92 ± 0.06	0.037

#### 3.2.5 Subgroup Analysis for the Pixel Value Ratio Growth Value Based on the Involvement of the Internal Fixator During the Consolidation Period

A total of 38 and 50 subjects with or without the involvement of an internal fixator during the consolidation period were recruited for this analysis. There was no significant difference in the PVR growth value between the subjects with and without the involvement of the internal fixator (0.04 ± 0.04 vs. 0.04 ± 0.04; *p* = 0.422) (Table 6).

**TABLE 6:** Subgroup analysis for the PVR growth based on the involvement of the internal fixator during the consolidation period.

	Internal fixator		*p*-value
	Involved (*n* = 38)	Uninvolved (*n* = 50)	
PVR growth value (mean ± SD)	0.04 ± 0.04	0.04 ± 0.04	0.422

### 3.3 Associations of the Healing Index and Lengthening Index With the Early Pixel Value Ratio of the Callus

A total of 113 patients were recruited for HI analysis, whereas 115 patients were employed for LI analysis. The average HI was 44.98 ± 49.44 days per centimeter, and the LI was 0.78 ± 0.77 months per centimeter, respectively. The results showed that the early PVR of the regenerated callus in the first month after osteotomy was moderately inversely associated with the HI (r = −0.211; *p* = 0.029) and LI (r = −0.210; *p* = 0.029) (Table 7).

**TABLE 7:** Associations of the healing index and lengthening index with the early PVR value.

PVR value	Index	
	Healing index (*n* = 113)	Lengthening index (*n* = 115)
First month	r = −0.211; *p* = 0.029	r = −0.210; *p* = 0.029
Second month	r = −0.125; *p* = 0.210	r = −0.191; *p* = 0.053
Third month	r = 0.026; *p* = 0.801	r = −0.017; *p* = 0.867

## 4 Discussion

**FIGURE 4:**
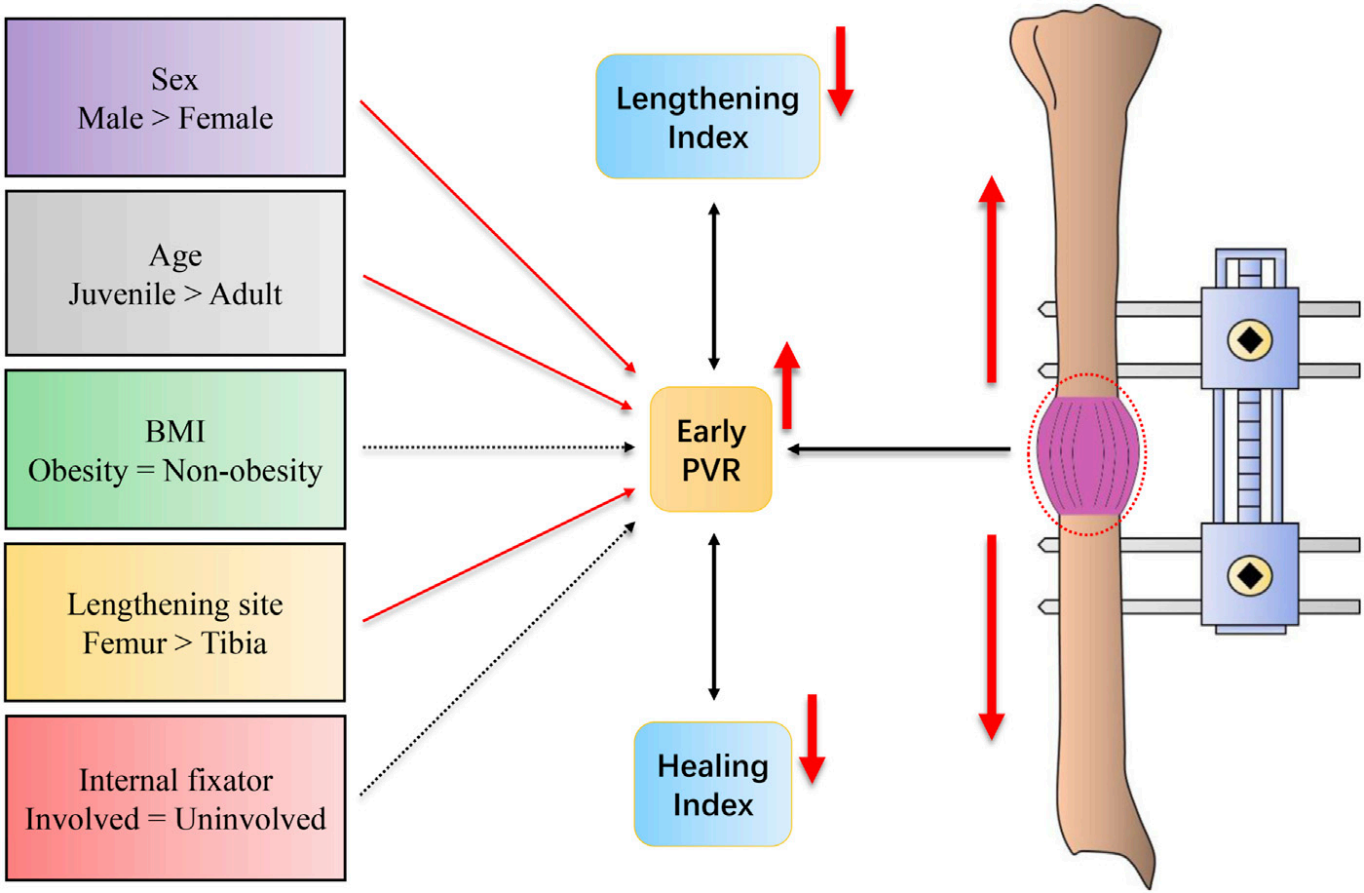
Schematic diagram for the results of this study.

Our results showed that the early PVR is gradually increasing in the first 3 months after osteotomy, and the early PVRs in juvenile, male, and femur sites are significantly higher than those in adult, female, and tibial site subjects. Moreover, the early PVR is moderately inversely associated with the HI and LI, respectively (Figure 4).

Generally speaking, the PVR is mainly utilized to assess the maturity of the late bone callus in order to identify the time to remove the external fixator. Zhao et al. (2009) demonstrated that the PVR could be served as an objective parameter for callus measurement, which provided guidance for the timing of external fixator removal. Bafor et al. found that there were no adverse effects when subjects commenced full weight-bearing when three out of the four cortices of the anteroposterior and lateral radiographs had a PVR of 0.93. Moreover, both Song et al. (2012) and Vulcano et al. (2018) indicated that the PVR could be utilized as a criterion for callus maturation and full weight-bearing. On this basis, our study further analyzed the early PVR of the callus during DO and its potential influencing factors and association with HI and LI.

Interestingly, Koczewski and Shadi (2013) found that the LI was increased with aging, and the LI for the femur was significantly lower than that for the tibia. Indeed, the bone metabolism system with an equilibrium of bone resorption and formation changes with aging (Kloss and Gassner, 2006), and the arterial blood supply of the femur is also richer than that of the tibia (Kizilkanat et al., 2007) (the nutrient artery is the main blood supply to the long bone). Moreover, Mehta et al. (2011) found that being female was an independent risk factor for bone healing *in vivo*, which may be attributed to a reduction in the mesenchymal stem cell quantity in the bone marrow (Strube et al., 2009). Consistently, we did find that the early PVRs in male, juvenile, and femur sites were significantly higher than those in female, adult, and tibial site subjects, respectively. On the other hand, obesity may induce ectopic adipocyte accumulation in bone marrow cavities, which is considered to impair osteogenic regeneration (Ambrosi et al., 2017). Furthermore, Sun et al. (2011) found more favorable progress in callus regeneration during bone lengthening with the involvement of an internal fixator. Nevertheless, no significant difference in the PVR with regard to BMI and the involvement of the internal fixator was identified in our study. It was speculated that the PVR might not be sensitive enough to reflect the issues. More importantly, the number of obese subjects is rather small, which may inevitably influence our results. Basically, a series of clinical issues caused by an external fixator (for example, inconvenient activities, psychological impact, and uncomfortableness) (Castelein and Docquier, 2016; Nguyen Van and Le Van, 2021) may be avoided by the involvement of an internal fixator. However, our results showed that the early PVR value might not reflect the clinical benefit of an internal fixator (additional medical resources are also consumed). Therefore, the involvement of the internal fixator in bone lengthening still needs to be discussed further. In addition, it is well known that both the LI and HI indicate the bone healing potential (Koczewski and Shadi, 2013; Wright et al., 2020). Our study suggests that the early PVR is moderately inversely associated with LI and HI, which may partly reflect the potential clinical outcome of bone lengthening. However, our results are restricted to the nature of the retrospective design. Taken together, further large well-designed prospective studies are still needed.

The advantages of this study are as follows: first, this is the first study to assess the early PVR value and its potential influencing factors (sex, chronological age, and lengthening site) in bone lengthening. Second, the associations of the HI and LI with the early PVR were also discussed first. Third, this is the largest sample-sized study for PVR analysis until now (others only involved tens of subjects). Fourth, our results may provide the potential clinical value of the early PVR in subjects with bone lengthening. The limitations to the present study should also be acknowledged. First, several issues cannot be addressed due to the nature of the retrospective study design. Second, due to the limited available evidence, bone nonunion cannot be considered in the present study. Third, the disturbance by metal fixtures during the PVR measurement may still slightly influence the PVR assessment. Fourth, some data for BMI and PVR growth were lost in our study, which leads to a different sample size between overall and subgroup analysis. Fifth, the disuse osteopenia of the adjacent bone caused by DO has been ignored in our study. Last but not the least, the number of obese subjects is relatively small in our study.

Our results showed that the early PVR is gradually increasing in the first 3 months after osteotomy, which may be significantly influenced by chronological age, sex, and lengthening site. Moreover, the early PVR of the callus may reflect the potential clinical outcome for DO. Our results may be beneficial to the clinical management of the subjects with bone lengthening.

## Data Availability

The original contributions presented in the study are included in the article/supplementary material; further inquiries can be directed to the corresponding authors.
